# A Lightweight White-Box Symmetric Encryption Algorithm against Node Capture for WSNs †

**DOI:** 10.3390/s150511928

**Published:** 2015-05-21

**Authors:** Yang Shi, Wujing Wei, Zongjian He

**Affiliations:** School of Software Engineering, Tongji University, No.4800 Cao’An Highway, Shanghai 201804, China; E-Mails: shiyang@tongji.edu.cn (Y.S.); clrowd@gmail.com (W.W.)

**Keywords:** wireless sensor networks, white-box attack contexts, node capture, symmetric encryption algorithms

## Abstract

Wireless Sensor Networks (WSNs) are often deployed in hostile environments and, thus, nodes can be potentially captured by an adversary. This is a typical white-box attack context, *i.e*., the adversary may have total visibility of the implementation of the build-in cryptosystem and full control over its execution platform. Handling white-box attacks in a WSN scenario is a challenging task. Existing encryption algorithms for white-box attack contexts require large memory footprint and, hence, are not applicable for wireless sensor networks scenarios. As a countermeasure against the threat in this context, in this paper, we propose a class of lightweight secure implementations of the symmetric encryption algorithm SMS4. The basic idea of our approach is to merge several steps of the round function of SMS4 into table lookups, blended by randomly generated mixing bijections. Therefore, the size of the implementations are significantly reduced while keeping the same security efficiency. The security and efficiency of the proposed solutions are theoretically analyzed. Evaluation shows our solutions satisfy the requirement of sensor nodes in terms of limited memory size and low computational costs.

## 1. Introduction

Wireless Sensor Networks (WSNs) are often deployed in hostile environments such as wide forests and public parking lots. In addition, data are transmitted using wireless networks over the air. Therefore, security measures such as how to prevent eavesdropping of private information are critical. Furthermore, the sensor nodes are also subject to be captured and surreptitiously used by an adversary [1,2]. If a WSN node is captured by an adversary, the adversary can then easily extract cryptographic primitives and obtain unlimited access to the information stored in the node’s memory chips, with the potential to cause substantial damage to the entire system. This process can be achieved by using reverse engineering followed by probing techniques that require access to the chip level components of the device [3,4].

Symmetric encryption is one of the most important cryptographic primitives. Unfortunately, the standard design and implementation of symmetric encryption algorithms are not intended to be applied in environments where their execution could be observed. In fact, standard cryptographic models assume that endpoints (e.g., hosts or sensor nodes) can be fully trusted. However, if the endpoints are deployed in potentially hostile environments and are captured, the cryptographic keys may be directly visible to the attackers. By actively monitoring standard cryptographic functions or memory dumps, attackers are able to extract the keys. This is a critical security risk for the WSN system. To build a secure system based on WSNs, we must come up with a countermeasure against the threat of node capture.

From the viewpoint of security research, an outdoor WSN node captured by an attacker is in a typical white-box attack context (WBAC). As is well known, secure computing in a white-box attack context is very challenging, because WBAC assumes that fully-privileged attackers share the same host with cryptographic software, and have complete access to the implementation of the cryptographic algorithms. What is worse, dynamic execution (with instantiated cryptographic keys) can also be observed; and the internal details of cryptographic algorithms are completely visible and alterable [5,6].

The objective of this work is to design a novel lightweight symmetric encryption algorithm for wireless sensor networks against node capture attacks. With the help of our algorithm, even though the sensor nodes are captured by an adversary, *i.e.*, in a typical white-box context, the cryptographic keys are still safe and cannot be compromised.

In recent years, researchers have proposed some white-box encryption algorithms that intend to provide practical protection for software implemented on a non-trustable host. However, they cannot be directly applied for WSN nodes. This is because existing algorithms have strong requirements in terms of memory footprint and computation power. Unfortunately, the sensor nodes in WSN are a typical resource-constrained environment. The limited memory and CPU resource cannot afford to run the existing algorithms. For example, almost all of existing white-box encryption algorithms, such as [5,6,7,8,9], require at least 752 KB of memory to store lookup tables, but the size of the internal memory of a node is usually only 512 KB or even less, which is a crucial restriction of white-box encryption algorithms. To the best of our knowledge, there is only one published white-box encryption algorithm with small size of lookup table [10]. It needs only about 148.625 KB to store the static data. However, this white-box encryption algorithm can still be improved in both security and complexity.

Motivated by the security challenge of node capture on resource limited nodes of sensor networks, we provide a lightweight white-box encryption algorithm for symmetric cryptography primitives to prevent node capture attacks. The white-box encryption algorithm can maintain a relatively high security level in white-box attack contexts.

The design objectives of our algorithm are as follows:

● Low memory requirements.
● Low computational costs.
● Node-compromise resilience.

The contribution of this paper can be summarized as follows:

We propose a class of white-box encryption algorithms that obfuscates the block cipher SMS4, which is immune from various attack methods in the black-box model. Secondly, new obfuscation techniques are used to enhance the difficulty of attack. Therefore, our algorithms are also immune from the three known effective attack methods [11,12,13] against white-box encryption algorithms based on the substitution permutation network. Finally, intensive security analysis and measurement of the proposed algorithms are also provided.

The remainder of this paper is organized as follows: First, a brief review of existing white-box encryption algorithms is presented in Section 2. Then, the design of a new white-box symmetric encryption algorithm is provided in Section 3. The time complexity, size and security of our algorithm are then analyzed in Section 4. Two methods for further improvement of the white-box SMS4 are discussed in Section 5. In Section 6, we compare the proposed algorithms with existing ones to demonstrate its advantages. In Section 7, we analyze why the proposed algorithms are secure against white-box attacks and side-channel attacks. Finally, the article concludes with a discussion of the findings. Note that the terms “white-box encryption algorithm” and “white-box implementation of an encryption algorithm” are used interchangeably throughout the paper.

Note that this paper is an extended version of [14]. A summary of differences of this paper and the previous version is as follows.

**Figure 1:**
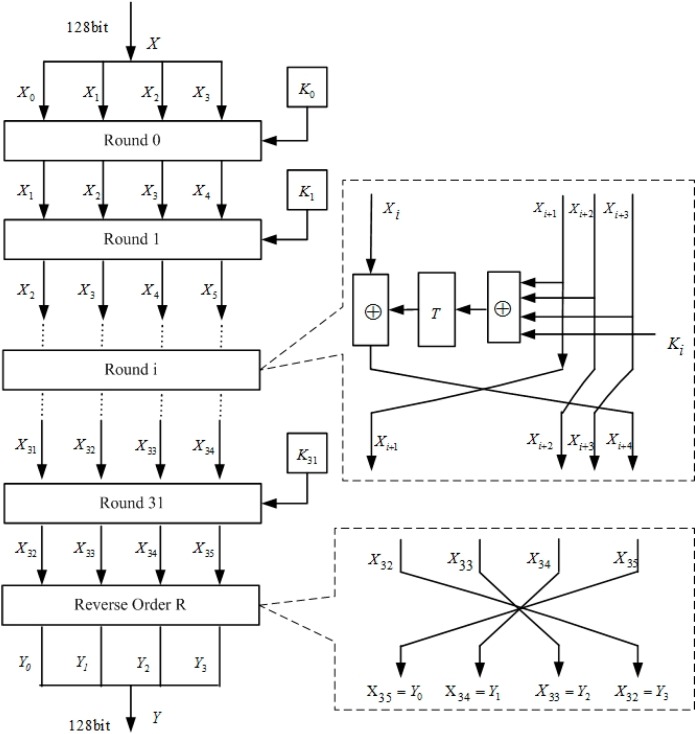
The flow and structure of SMS4.

**Figure 2:**
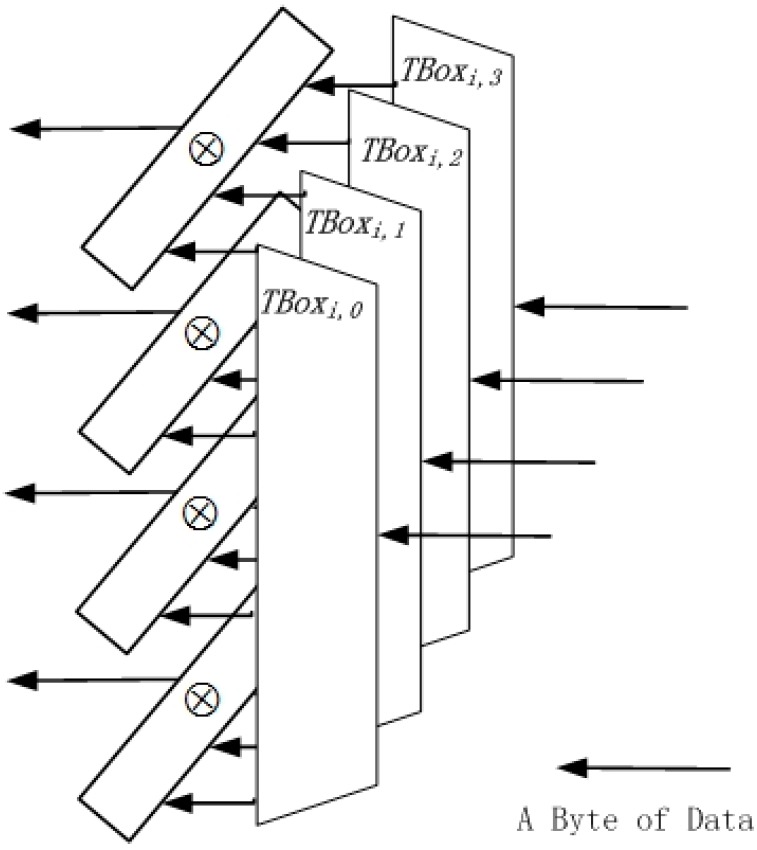
The structure of T-Boxes in a round.

**Figure 3:**
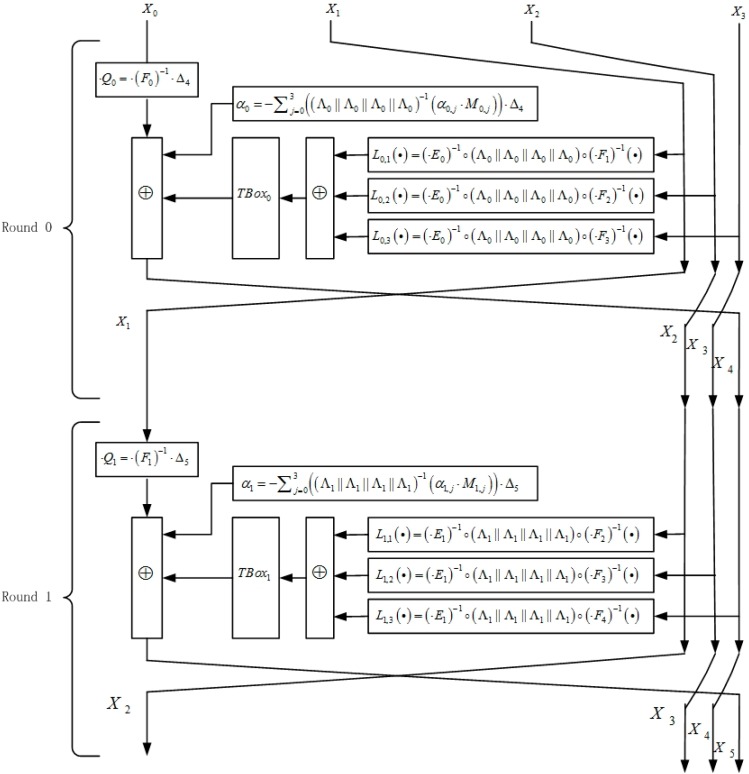
The structure of round 0 and round 1.

**Figure 4:**
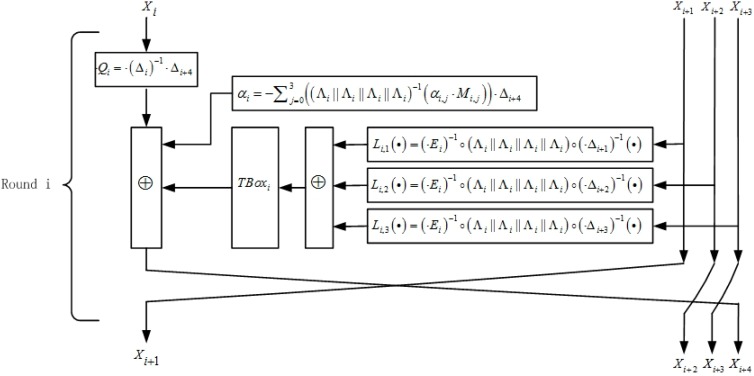
The structure of an intermediate round.

(1) The (conference version of the) white-box SMS4 algorithm is slightly revised to improve the performance.
(2) Two new methods on further improve the white-box SMS4 are provided in Section 5. One is about security-efficiency trade-off and an aggressive implementation for performance sensitive scenarios, the other is about a strong implementation using non-standard S-Boxes for security sensitive scenarios. The strong white-box SMS4 is immune from all known attacks and possible adaptations.
(3) A new section “7. Security against white-box attacks and side-channel attacks” is added. Analyses on security against known white-box attacks are extended, especially on an attack that is published after the conference paper is accepted. Analyses on security against side-channel attacks are included in this version.
(4) To further explain existing research on design and implementation of white-box encryption algorithms, we added a new section “2. Review on White-box Encryption Algorithms”. Results of corresponding cryptanalysis are also presented in this section.
(5) In Section 3, we fleshed out the description of the white-box SMS4 algorithm. Some figures (Figure 1, Figure 2, Figure 3 and Figure 4) are provided to make the description more clear than the conference version.
(6) Comparisons with other methods are extended and refined.

## 2. Review of White-Box Encryption Algorithms

Existing research on white-box cryptography has focused on white-box implementations of classical symmetric encryption algorithms, such as DES and AES.

Chow *et al.* [5] proposed a white-box implementation of DES by interleaving affine transformations and using de-linearization techniques. Chow *et al*. implemented white-box AES [6] by representing it with a set of key-dependent look-up tables. They suggested the use of these two white-box encryption algorithms in DRM applications to protect digital information content and the associated usage rights from unauthorized access, use, and dissemination. These two works form the foundation of almost all white-box encryption papers. Many attacks have occurred against the white-box encryption algorithms proposed in [5,6], and these two algorithms are insecure now. The next two paragraphs discuss the attacks on [5,6].

Jacob *et al.* [15] proposed a fault injection based attack, where an attacker injects errors into the environment during program execution, to defeat some obfuscation methods. They presented a cryptanalysis of the naked variant of the Chow *et al*.’s white-box DES, that is, a variant without external encodings. Similar to Chow *et al*.’s white-box DES, Link *et al*. [7] implemented white-box DES and white-box triple-DES algorithms with alterations that improved the security of the key. Their algorithms are secure against the previously published attacks on Chow *et al*.’s white-box DES implementation and their own adaptation of a statistical bucketing attack. In 2007, Wyseur *et al*. [16] and Goubin *et al.* [17], independently of each other, broke all existing obfuscation methods of DES. These attacks were based in a truncated differential cryptanalysis. Goubin *et al.* presented an attack that analyzed the first round of the white-box DES implementations, while Wyseur *et al*. presented an attack that works on the internal information. Hence, none of proposed white-box DES implementations are secure.

Billet *et al.* [11] presented an efficient practical attack against the obfuscated AES implementation proposed by Chow *et al*., with negligible memory and worst work factor of 2^30^. In 2009, Michiels *et* *al*. [12] generalized the attack that could be deployed on a generic class of white-box implementations. One of the most important design purposes of the proposed algorithms is to protect the white-box cipher against attacks in [11,12]. The most time-consuming part of Billet *et al.*’s attack [11] is finding the used byte permutation up to an affine mapping, which takes a work factor of 2^24^ in the worst situation. In 2012, Tolhuizen [18] provided a variation on this part of the attack, reducing the work factor to at most 2^14^. With this improvement, the overall worst work factor of breaking Chow *et al*.’s white-box AES in [6] is reduced from 2^30^ to 2^20^. 

The two key factors of a white-box encryption algorithm are size and security. Unfortunately, in many cases, the two key factors are a tradeoff and cannot be achieved simultaneously. Therefore, some recent implementations only focus on one different key factor. For size consideration, Shi *et al.* [19] proposed a white-box encryption algorithm for computing using a mobile agent protected with time-limited black box security [20]. The size of this implementation is small and suitable for migrating from one host to another as a part of a mobile agent. For secure consideration, Xiao *et al.* [8] proposed a white-box AES after a detailed analysis of attack techniques in [11]. The size of this implementation is considerably large to achieve a higher security level. In Xiao *et al*.’s scheme, the obfuscation works on at least two cells of an AES state; moreover, the attacker cannot divide it into smaller units (e.g., one cell of an AES state) and remove it using the attack techniques proposed in [11]. The time complexity of the Xiao-Lai white-box AES implementation is O(2^24^), which is slower than the Chow *et al*.’s implementation in [6] (O(2^20^)), and the size is 20,502 KB. 

De Mulder *et al*. (2012) presented a practical cryptanalysis of Xiao *et al*.’s white-box AES in [13]: they applied the linear equivalence algorithm presented by Biryukov *et al*. [21] as a building block in their key-extraction algorithm. The cryptanalysis efficiently extracts the AES key with a work factor of about 2^32^.

Another white-box implementation of AES was proposed by Karroumi in 2011 [9]. This implementation makes InvSubBytes and InvMixColumns operations variable by using additional sets of coefficients taken from dual representations of AES. Karroumi claimed that the expected security level is raised from 2^30^ − 2^91^. However, an algebraic analysis [22] was proposed in 2013 and Karroumi’s implementation can be easily broken.

A white-box SMS4 algorithm is proposed by Xiao *et al.* in [10]. However, in 2013, Lin *et al*. [23] proposed an efficient attack that can extract the round key embedded in Xiao *et al*.’s white box SMS4 implementation, with worst work factor 2^47^. In this paper, we follow the thread of Xiao *et al*.’s white-box SMS4 to a certain extent, and some obfuscation transformations similar to transformations in [10] are also used. Differently, we use isomorphic transformations and even special substitution components to achieve a higher security level. Further, in the strong version, randomly generated non-standard S-Boxes are used to enhance the security.

With the recent development of attack techniques, the security of the white-box encryption algorithms, such as [5,6,7,8,9,10] has been challenged. Furthermore, most of them require a rather large memory to store lookup tables, but the size of internal memory of a node is usually only 512 KB or even less, which is also a crucial restriction of white-box encryption algorithms. Hence, different from existing solutions, we propose a white-box encryption algorithm that obfuscates the block cipher SMS4. The proposed algorithm tries to maximize the security level with the constraint of small data size.

## 3. A New White-Box SMS4 Encryption Algorithm

### 3.1. The SMS4 Block Cipher

SMS4 [24] is a Chinese national standard for block cipher, mandated for use in protecting wireless networks, and issued in January 2006. SMS4 is a 32 rounds unbalanced Feistel network (UFN); both the block and the key size are 128 bits. Encryption and decryption have the same structure except that the round key schedule for decryption is the reverse of the round key schedule for encryption.

The nonlinear part τ of a round transformation is defined as follow:

Let

(1) $$A=\left(a_{0},a_{1},a_{2},a_{3}\right)\in GF\left(2^{8}\right)$$

(2) $$B=\left(b_{0},b_{1},b_{2},b_{3}\right)\in GF\left(2^{8}\right)$$

(3) $$\left(b_{0},b_{1},b_{2},b_{3}\right)=\;\tau \left(A\right)=\left(Sbox\left(a_{0}\right),Sbox\left(a_{1}\right),Sbox\left(a_{2}\right),Sbox\left(a_{3}\right)\right)$$

The linear part $L:GF{\left(2\right)}^{32}\to GF{\left(2\right)}^{32}$ of a round transformation is a linear mapping as follow:

(4) C=L(B)=B⊕(B<<<2)⊕(B<<<10)⊕(B<<<18)⊕(B<<<24)

Let $K_{i}$ be the round key of the *i*-th round. The round function R is defined as follow:

(5) $$R:{\left(GF{\left(2\right)}^{32}\right)}^{5}\to GF{\left(2\right)}^{32};R\left(X_{i},X_{i+1},X_{i+2},X_{i+3},K_{i}\right)=X_{i}⊕T\left(X_{i+1}⊕X_{i+2}⊕X_{i+3}⊕K_{i}\right)$$

where T=L◦τ.

The flow and structure of SMS4 encryption are illustrated in Figure 1.

### 3.2. Components of the White-Box Encryption Algorithm

To hide the encryption key, we merge several steps of each round function of SMS4 into table lookups blended by randomly generated mixing bijections. In this section, we investigate how to design such tables and how randomly generated mixing bijections can be counteracted. We use techniques from [10] and [9] to obtain the obfuscated implementation. To enhance the security level, following design strategies are used.

(1) Distinct representations of the cipher, especially the S-Box, are used in every T-Box table. Hence, we get more than $2^{13}$ times work factor than when only using the standard representation.
(2) External encodings are used to protect the first round and the last round. Otherwise these two rounds were ‘naked’ and cast effect would help an attacker to break the white-box implementation more easily.
(3) We transform the output mixing mappings of T-Box tables from linear mappings into affine mappings. This transformation would offer $2^{8}$ times work factor with the cost of 32 times 32-bit exclusive or (XOR).

Furthermore, we reduce the number of matrix multiplications used in the encryption process. This would clearly increase the speed of encryption.

Our design is partially based on Liu *et al*.’s analysis of the SMS4 block cipher [25]. They have shown that the S-Box of SMS4 is of the form $S\left(x\right)\;=\;I\left(x\cdot A_{1}+C_{1}\right)\cdot A_{2}+\;C_{2}$ with $A_{1},A_{2}\in GL\left(8,2\right)$ and $C_{1},C_{2}\in GF{\left(2\right)}^{8}$. Their experiments finally found that the irreducible polynomial is $f\left(x\right)=x^{8}+x^{7}+x^{6}+x^{5}+x^{4}+x^{2}+1$. The values of $C_{1},C_{2}$ and $A_{1},A_{2}$ are shown in Equations (6) and (7).

(6) $$A_{1}=A_{2}=\left(\begin{array}{l} \text{1 1 1 0 0 1 0 1}\\ \text{1 1 1 1 0 0 1 0}\\ \text{0 1 1 1 1 0 0 1}\\ \text{1 0 1 1 1 1 0 0}\\ \text{0 1 0 1 1 1 1 0}\\ \text{0 0 1 0 1 1 1 1}\\ \text{1 0 0 1 0 1 1 1}\\ \text{1 1 0 0 1 0 1 1} \end{array}\right)$$

(7) $$C_{1}=C_{2}=\;(1,\;1,\;0,\;0,\;1,\;0,\;1,\;1)$$

Hence, SMS4 is an $EGF(2^{8})$ cipher [26]. For each irreducible polynomial, we can define its 8 square dual ciphers. Since there are 30 irreducible polynomials, we get that there are 240 dual ciphers for a SMS4 cipher. Furthermore, Raddum presented 9120 other representations of $GF(2^{8})$ [27] to construct more duals of AES. Similarly, more dual SMS4 ciphers can also be obtained by these representations.

Let $R=\left\{r_{0},r_{1},\cdots ,r_{9359}\right\}$ be the set of all these 9360 representations. For each i=0,1,⋯,31, $\Lambda_{i}$ is a mapping which transforms the SMS4 cipher in representation $r_{0}$ to a dual SMS4 cipher in representation $r_{j_{i}}$ where $j_{i}\overset{\$}{\leftarrow }\left[1,2,\cdots ,9359\right]$.

Let $F_{i},i=0,1,2,3$, $\Delta_{i},i=0,1,\cdots ,35$ and $G_{i},i=0,1,2,3$ be randomly generated 32 × 32 nonsingular matrixes over GF(2). For i=0,1,2,3, $\Delta_{i}=F_{i}$.

Let M be the matrix representation of the linear transformation L and suppose $M=\left[M_{0},M_{1},M_{2},M_{3}\right]$ where $M_{0},M_{1},M_{2},M_{3}$ are four 8 × 32 binary matrixes.

The substitution transformation $S_{i}$ is given by Equation (8).

(8) $$S_{i}:GF{\left(2\right)}^{8}\to GF{\left(2\right)}^{8},x\mapsto \Lambda_{i}\left(Sbox\left({\Lambda_{i}}^{-1}\left(x\right)\right)\right)$$

Let $x\in GF{\left(2\right)}^{8},y\in GF{\left(2\right)}^{32}$, the T-Box lookup table with index <*i*,*j*> , *i.e*., $TBox_{i,j}$, is defined by Equation (9).

(9) $$y=TBox_{i,j}\left(x\right)=\left({\left(\Lambda_{i}||\Lambda_{i}||\Lambda_{i}||\Lambda_{i}\right)}^{-1}\left(\left(S_{i}\left(K_{i,j}+x\cdot E_{i,j}\right)+\alpha_{i,j}\right)\cdot M_{i,j}\right)\right)\cdot \Delta_{i+4}$$

where $\Lambda_{i}||\Lambda_{i}||\Lambda_{i}||\Lambda_{i}$ refers to four $\Lambda_{i}$ operating in parallel.

Components in Equation (9) are defined as follows.

$\alpha_{i,j}$ is a randomly generated element of $GF{\left(2\right)}^{8}$.

$E_{i,j}$ is a randomly generated 8 × 8 nonsingular matrix over GF(2).

$K_{i,j}=\Lambda_{i}\left(I_{4,j}\left(K_{i}\right)\right)$, where $K_{i}$ is the *i*-th round key.

$M_{i,j}$ is a 8 × 32 matrix corresponds to the linear transformation $\tau_{i,j}$ that is defined in Equation (10).

(10) $$\tau_{i,j}:GF{\left(2\right)}^{8}\to GF{\left(2\right)}^{32};x\mapsto \left(\Lambda_{i}||\Lambda_{i}||\Lambda_{i}||\Lambda_{i}\right)\left(\left({\Lambda_{i}}^{-1}\left(x\right)\right)\cdot M_{j}\right)$$

For each i, $TBox_{i}$ is a bijection from $GF{\left(2\right)}^{32}$ to $GF{\left(2\right)}^{32}$. Let $x_{j}\in GF{\left(2\right)}^{8},j=0,1,2,3$, $TBox_{i}$ is defined in Equation (11).

(11) $$TBox_{i}=\sum_{j=0}^{3}TBox_{i,j}\left(x_{j}\right)$$

The structure that is shown in Figure 2 depicts the usage of T-Boxes in a round.

Furthermore, in each round, $\alpha_{i}$, $L_{i,n}$ and $Q_{i}$ are defined as in Equations (12)–(14).

(12) $$\alpha_{i}=-\sum_{j=0}^{3}\left({\left(\Lambda_{i}||\Lambda_{i}||\Lambda_{i}||\Lambda_{i}\right)}^{-1}\left(\alpha_{i,j}\cdot M_{i,j}\right)\right)\cdot \Delta_{i+4}$$

(13) $$Q_{i}=\left({\Delta_{i}}^{-1}\right)\cdot \Delta_{i+4}$$

(14) $$L_{i,n}=\left(\cdot {E_{i}}^{-1}\right)◦\left(\Lambda_{i}||\Lambda_{i}||\Lambda_{i}||\Lambda_{i}\right)◦\left(\cdot {\Delta_{i+n}}^{-1}\right),n=1,2,3$$

where ${E_{i}}^{-1}$ is given by Equation (15).

(15) $${E_{i}}^{-1}=diag\left\{{E_{i,0}}^{-1},{E_{i,1}}^{-1},{E_{i,2}}^{-1},{E_{i,3}}^{-1}\right\}$$

This ends the description of components.

The round function of our white-box implementation is:

(16) $$\begin{array}{l} R_{i}:{\left(GF{\left(2\right)}^{32}\right)}^{4}\to GF{\left(2\right)}^{32};\\ R_{i}\left(X_{i},X_{i+1},X_{i+2},X_{i+3}\right)=\;\alpha_{i}+X_{i}\cdot Q_{i}+\sum_{j=0}^{3}TBox_{i,j}\left(I_{4,j}\left(\sum_{n=1}^{3}L_{i,n}\left(X_{i+n}\right)\right)\right) \end{array}$$

Figure 3 and Figure 4 show the structure of the first two rounds and an intermediate round, respectively.

### 3.3. The Complete White-Box Encryption Algorithm

Now, using the components provided in the previous subsection, the white-box encryption algorithm is described as follows (Algorithm 1):
**Algorithm 1**  *SMS*4_*W*_[*K*] (*on input X*):(1)  (*X*_0_, *X*_1_, *X*_2_, *X*_3_) ← *X*(2)  *i* ← 0(3)    *n* ← 1(4)      *Z_n_* ← *L_i,n_* (*X_i_*_+_*_n_*)(5)      *n* ← *n* + 1(6)      *if* (*n* <= 3) *goto*(4); *else* *goto*(7)(7)    *Z* ← *Z*_1_ ⊕ *Z*_2_ ⊕ *Z*_3_(8)    *X_i_*_+4_ ← *TBox_i_* (*Z*) ⊕ *α_i_* ⊕ (*X_i_* ∙ *Q_i_*)(9)    *i* ← *i* + 1;(10)     *if* (*i* < 32)  *goto*(3) *else* *goto*(11)(11)  *Y* ← (*X*_32_, *X*_33_, *X*_34_, *X*_35_)(12)  *output* *Y*

Let

(17) $$F:{\left(GF{\left(2\right)}^{32}\right)}^{4}\to {\left(GF{\left(2\right)}^{32}\right)}^{4};F\left(X\right)=\left(X_{0},X_{1},X_{2},X_{3}\right)\cdot \left(\begin{array}{cccc} F_{0} & & & \\ & F_{1} & & \\ & & F_{2} & \\ & & & F_{3} \end{array}\right)$$

and

(18) $$G:{\left(GF{\left(2\right)}^{32}\right)}^{4}\to {\left(GF{\left(2\right)}^{32}\right)}^{4};G\left(X\right)=\left(X_{0},X_{1},X_{2},X_{3}\right)\cdot {\left(\begin{array}{cccc} & & & G_{3}\\ & & G_{2} & \\ & G_{1} & & \\ G_{0} & & & \end{array}\right)}^{-1}$$

where $X=\left(X_{0},X_{1},X_{2},X_{3}\right)$ and $G_{k}={\Delta_{32+k}}^{-1},k=0,1,2,3$.

Now, instead of SMS4[K], $SMS4_{W}[K]=G^{-1}◦SMS4[K]◦F^{-1}$ is implemented, where F and G are external input and output encodings. When ciphertext encrypted by $SMS4_{W}[K]$ needs to be decrypted, one should only apply $SMS{4_{W}}^{-1}[K]=F◦SMS4^{-1}[K]◦G$.

The following proposition shows the correctness of our algorithm.

**Proposition 1.** *The encryption algorithm* $SMS4_{W}[K]$ *is such that*

(19) $$G◦SMS4_{W}[K]◦F=SMS4[K]$$

**Proof.**

Let $X=\left(X_{0},X_{1},X_{2},X_{3}\right),X_{i}\in GF{\left(2\right)}^{8},i=0,1,2,3$ be the input of the first round of $SMS4_{W}[K]◦F$. Then

$$\begin{array}{l} R_{0}◦F\left(X\right)\\ =R_{0}\left(F\left(X_{0},X_{1},X_{2},X_{3}\right)\right)\\ =\;\alpha_{0}+\left(X_{0}\cdot F_{0}\right)\cdot Q_{0}+\sum_{j=0}^{3}TBox_{0,j}\left(I_{4,j}\left(\sum_{n=1}^{3}L_{0,n}\left(X_{n}\cdot F_{n}\right)\right)\right)\\ =\;\alpha_{0}+\left(X_{0}\cdot F_{0}\right)\cdot Q_{0}+\sum_{j=0}^{3}TBox_{0,j}\left(I_{4,j}\left(\sum_{n=1}^{3}\left(\begin{array}{l} \left(\Lambda_{0}||\Lambda_{0}||\Lambda_{0}||\Lambda_{0}\right)\\ \left(X_{n}\cdot F_{n}\cdot {\Delta_{0+n}}^{-1}\right) \end{array}\right)\cdot {E_{0}}^{-1}\right)\right)\\ =\;\alpha_{0}+\left(X_{0}\cdot F_{0}\right)\cdot Q_{0}+\sum_{j=0}^{3}TBox_{0,j}\left(I_{4,j}\left(\sum_{n=1}^{3}\left(\left(\Lambda_{0}||\Lambda_{0}||\Lambda_{0}||\Lambda_{0}\right)\left(X_{n}\right)\cdot {E_{0}}^{-1}\right)\right)\right)\\ =\;\alpha_{0}+\left(X_{0}\cdot F_{0}\right)\cdot Q_{0}+\sum_{j=0}^{3}\left(\begin{array}{l} \left({\left(\Lambda_{0}||\Lambda_{0}||\Lambda_{0}||\Lambda_{0}\right)}^{-1}\left(\alpha_{0,j}\cdot M_{0,j}\right)\right)\cdot \Delta_{4}+\\ \left({\left(\Lambda_{0}\right)}^{-1}S_{0}\left(K_{i,j}+\Lambda_{0}\left(I_{4,j}\left(X_{1}+X_{2}+X_{3}\right)\right)\right)\right)\cdot M_{0,j}\cdot \Delta_{4} \end{array}\right)\\ =\;\alpha_{0}+\left(X_{0}\cdot F_{0}\right)\cdot Q_{0}+\sum_{j=0}^{3}\left(\begin{array}{l} \left({\left(\Lambda_{0}||\Lambda_{0}||\Lambda_{0}||\Lambda_{0}\right)}^{-1}\left(\alpha_{0,j}\cdot M_{0,j}\right)\right)\cdot \Delta_{4}+\\ \left({\left(\Lambda_{0}\right)}^{-1}\Lambda_{0}\right)\left(Sbox\left({\Lambda_{0}}^{-1}\left(K_{i,j}+\Lambda_{0}\left(I_{4,j}\left(\begin{array}{l} X_{1}+\\ X_{2}+X_{3} \end{array}\right)\right)\right)\right)\right)\\ \cdot M_{0,j}\cdot \Delta_{4} \end{array}\right)\\ =\;\alpha_{0}+\left(X_{0}\cdot F_{0}\right)\cdot Q_{0}+\sum_{j=0}^{3}\left(\begin{array}{l} \left({\left(\Lambda_{0}||\Lambda_{0}||\Lambda_{0}||\Lambda_{0}\right)}^{-1}\left(\alpha_{0,j}\cdot M_{0,j}\right)\right)\cdot \Delta_{4}+\\ \left({\left(\Lambda_{0}\right)}^{-1}\Lambda_{0}\right)\left(Sbox\left({\Lambda_{0}}^{-1}\left(\begin{array}{l} I_{4,j}\left(\Lambda_{0}\left(K_{i}\right)\right)+\\ \Lambda_{0}\left(I_{4,j}\left(X_{1}+X_{2}+X_{3}\right)\right) \end{array}\right)\right)\right)\\ \cdot M_{0,j}\cdot \Delta_{4} \end{array}\right)\\ =\;\alpha_{0}+\left(X_{0}\cdot F_{0}\right)\cdot Q_{0}+\sum_{j=0}^{3}\left(\begin{array}{l} \left({\left(\Lambda_{0}||\Lambda_{0}||\Lambda_{0}||\Lambda_{0}\right)}^{-1}\left(\alpha_{0,j}\cdot M_{0,j}\right)\right)\cdot \Delta_{4}+\\ \left(Sbox\left(I_{4,j}\left(K_{i}+X_{1}+X_{2}+X_{3}\right)\right)\cdot M_{j}\right)\cdot \Delta_{4} \end{array}\right)\\ =\;\left(X_{0}\cdot F_{0}\right)\cdot Q_{0}\text{-}\sum_{j=0}^{3}{\left(\Lambda_{0}||\Lambda_{0}||\Lambda_{0}||\Lambda_{0}\right)}^{-1}\left(\alpha_{0,j}\cdot M_{0,j}\right)\cdot \Delta_{4}\\ +\sum_{j=0}^{3}\left(\begin{array}{l} \left({\left(\Lambda_{0}||\Lambda_{0}||\Lambda_{0}||\Lambda_{0}\right)}^{-1}\left(\alpha_{0,j}\cdot M_{0,j}\right)\right)\cdot \Delta_{4}+\\ \left(Sbox\left(I_{4,j}\left(K_{i}+X_{1}+X_{2}+X_{3}\right)\right)\cdot M_{j}\right)\cdot \Delta_{4} \end{array}\right)\\ =\;X_{0}\cdot F_{0}\cdot Q_{0}+\sum_{j=0}^{3}\left(Sbox\left(I_{4,j}\left(K_{i}+X_{1}+X_{2}+X_{3}\right)\right)\cdot M_{j}\cdot \Delta_{4}\right)\\ =\;X_{0}\cdot \Delta_{4}+\sum_{j=0}^{3}\left(Sbox\left(I_{4,j}\left(K_{i}+X_{1}+X_{2}+X_{3}\right)\right)\cdot M_{j}\right)\cdot \Delta_{4}\\ =\;X_{0}\cdot \Delta_{4}+\left(Sbox||Sbox||Sbox||Sbox\right)\left(K_{i}+X_{1}+X_{2}+X_{3}\right)\cdot M\cdot \Delta_{4}\\ =\;\left(R\left(X_{0},X_{1},X_{2},X_{3},K_{0}\right)\right)\cdot \Delta_{4} \end{array}$$

where the round transformation R is defined in (5). We arrive at the last round by similar deductions on the previous rounds. 

The last round of $G◦SMS4_{W}[K]◦F$ works on the output of the previous round as follows:

$$\begin{array}{l} G\left(X_{32}\cdot \Delta_{32},X_{33}\cdot \Delta_{33},X_{34}\cdot \Delta_{34},R_{31}\left(X_{31}\cdot \Delta_{31},X_{32}\cdot \Delta_{32},X_{33}\cdot \Delta_{33},X_{34}\cdot \Delta_{34}\right)\right)\\ =\left(X_{32}\cdot \Delta_{32},X_{33}\cdot \Delta_{33},X_{34}\cdot \Delta_{34},X_{35}\cdot \Delta_{35}\right)\cdot {\left(\begin{array}{cccc} & & & G_{3}\\ & & G_{2} & \\ & G_{1} & & \\ G_{0} & & & \end{array}\right)}^{-1}\\ =\left(X_{32},X_{33},X_{34},X_{35}\right)\cdot \left[\begin{array}{cccc} & & & \Delta_{32}\cdot G_{3}^{-1}\\ & & \Delta_{33}\cdot G_{2}^{-1} & \\ & \Delta_{34}\cdot G_{1}^{-1} & & \\ \Delta_{35}\cdot G_{0}^{-1} & & & \end{array}\right]\\ =\left(X_{35},X_{34},X_{33},X_{32}\right) \end{array}$$

Hence

$G◦SMS4_{W}[K]◦F=SMS4[K]$.

This ends the proof.

## 4. Analysis of the Algorithm

### 4.1. Security Measurement in White-Box Attack Context

White-box diversity and white-box ambiguity are used by Chow *et al*. [5,6] to measure security strength of white-box encryption algorithms. These measurements are widely used in other related works such as [19] and [8]. In this section, the white-box diversity and white-box ambiguity of our algorithm will be analyzed, respectively.

The white-box diversity of a given component type is calculated by counting the number of distinct constructions that exist in a component of the same type, which measures variability among implementations and is useful in foiling pre-packaged attacks. For each T-Box table, the number of possible values of each round key is $2^{8}$. Since the possible number of nonsingular matrices of order *n* is $\left(2^{n}-1\right)\times \prod_{j=1}^{n-1}\left(2^{n}-1-\sum_{k=1}^{j}\left(\begin{array}{c} j\\ k \end{array}\right)\right)$, the possible number of $E_{i,j}$ is $2^{62}$ and the possible number of a strip of $\Delta_{i+4}$ is $2^{62\times 4}=2^{248}$. The possible number of $\alpha_{i,j}$ is $2^{8}$. Hence, the white-box diversity of a T-Box table is $2^{8}\times 2^{8}\times 2^{62}\times 2^{248}=2^{326}$. Similarly, the white-box diversity of a matrix-type component can be calculated. Due to the cask effect, only the lowest white-box diversity of all these components should be calculated. According to the description of the proposed algorithm, this value is $\omega \times 2^{992}=9360\times 2^{992}>2^{1005}$.

The white-box ambiguity of a component is obtained by counting the number of distinct constructions that produce exactly the same type of component. It measures the number of alternative interpretations or meanings of a specific component where an attacker must disambiguate in cracking one of the obfuscated cipher’s instances. The white-box ambiguity of a T-Box table is $2^{8}\times 2^{8}\times 2^{62}=2^{78}$. The lowest white-box diversity of all matrix-type components is ω=9360.

### 4.2. Size and Efficiency

There are three kinds of components that are used in the proposed algorithm: T-Box tables, 32 bit vectors and 32 × 32 binary matrixes.

The size of each T-Box table is $2^{8}\times 32$ bits = $2^{10}$ bytes = 1 KB. Every round needs four T-Boxes. Thus, the size of all 128 T-Box tables is 128 KB.

The size of a 32 × 32 binary matrix is $32\times 4=2^{7}$ bytes. For each i∈{0,1,⋯,31}, round i needs four 32 × 32 binary matrixes. The size of all these binary matrixes is 16 KB.

Furthermore, in every round there is a 32 bit value; all these values cost 128 bytes = 0.125 KB.

Therefore, the size of all the static data is 144.125 KB. It is smaller than that of all previously published white-box encryption algorithms. An extensive comparison is presented in the next section.

As to efficiency, 128 T-Box table lookups, 32 × 7 = 224 4-byte additions (exclusive or) and 128 matrix multiplications are needed in general. Look up a value in a T-Box table and 32 bit exclusive or is faster, but 32 × 32 binary matrix multiplication is time consuming. Compared to Xiao *et al*.’s implementation that needs 160 matrix multiplications, our implementation is much faster.

We can speed up the algorithm by trading memory for it. A multiplication table can map two input bytes ($a_{0},\cdots ,a_{7}$ and $b_{0},\cdots ,b_{7}$) into a single bit $\left(a_{0}\times b_{0}\right)⊕\left(a_{1}\times b_{1}\right)⊕\cdots ⊕\left(a_{7}\times b_{7}\right)$. With the help of such a multiplication table, we can optimize the efficiency of matrix multiplications and obtain a faster software implementation. The extra cost of memory is only 8 KB. This implementation requires four kinds of operations: multiplication table lookups, byte additions, (single) bit additions and T-Box table lookups.

**Table 1:** Number of operations in the fast software implementation of white-box SMS4.

Operation	Number of Operations	Formula
Multiplication table lookup	2^14^	128 × 32 × 4
Byte addition	0.875 × 2^10^	128 × 7
(single) Bit addition	3 × 2^12^	128 × 32 × 3
T-Box Table lookup	≈3 × 2^5^	32 × 3

In fact, the proposed algorithm running in the composite mode suggested by [28] is much faster than running in ECB mode.

To evaluate the size and computational efficiency of the proposed solution in real hardware, we have tested the performance of our algorithm on Intel iMote [29], a widely used sensor node in wireless sensor networks. We are planning to test the performance of our algorithm on more types of sensor nodes in the future.

## 5. Improvements of the Algorithm

In this section, we discuss two methods to further improve the white-box SMS4 that are introduced in Section 3. One is about security-efficiency trade-off and an aggressive implementation for performance sensitive scenarios, the other is a strong implementation using non-standard S-Boxes for security sensitive scenarios.

### 5.1. Security-Efficiency Trade-Off

The security of SMS4 in black-box attack contexts is rather satisfactory. Well-known results about black-box attacks against SMS4 are the linear and differential attacks against 22 rounds [30,31,32]. These attacks require $2^{117}$ known plaintexts and $2^{118}$ chosen plaintexts, respectively. In 2011, Su *et al.* proposed a differential cryptanalysis of 23-round SMS4 [33] with $2^{118}$ chosen plaintexts and $2^{126.7}$ encryptions. To the best of our knowledge, this is the best result. Rectangle and impossible differential attacks were studied in [34]. Algebraic and XLS attacks against reduced-round SMS4 have been studied in [35] and [36], respectively. None of these attacks can break the full round SMS4 cipher. We may aggressively estimate that a 24 round SMS4 encryption is sufficiently secure when being used in white-box attack contexts. For conservative users, they can choose the number of rounds between 25 and 32. Hence, when the white-box implementation is running in a resource-constrained device, such as a sensor node, we can make a security-efficiency trade-off by reducing the number of rounds. The 24 rounds white-box implementation is called “aggressive white-box SMS4 algorithm” in the rest of this paper. It is clear that the reduction of the rounds will not seriously influence the security level of our algorithm in white-box attack contexts.

**Figure 5:**
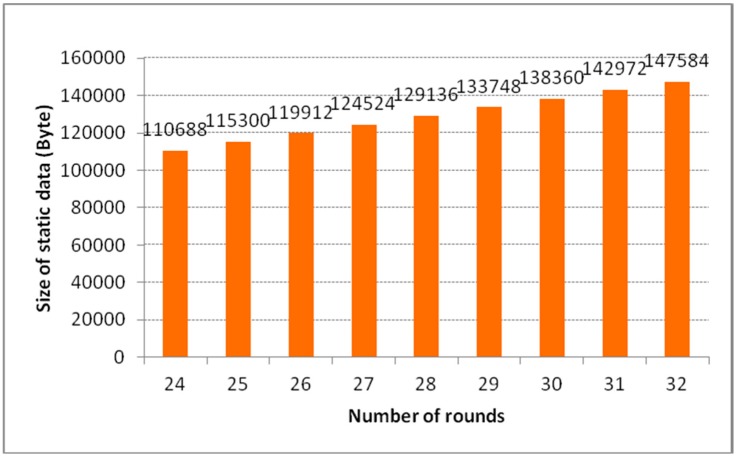
The size of static data.

**Figure 6:**
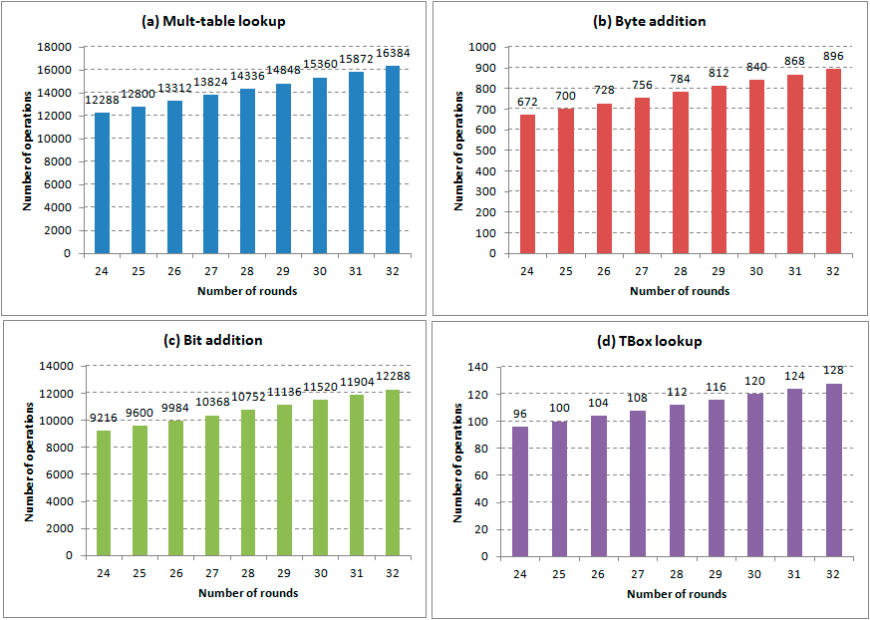
(**a**) The number of multi-table lookups; (**b**) The number of byte additions; (**c**) The number of bit additions; (**d**) The number of TBox lookups.

**Figure 7:**
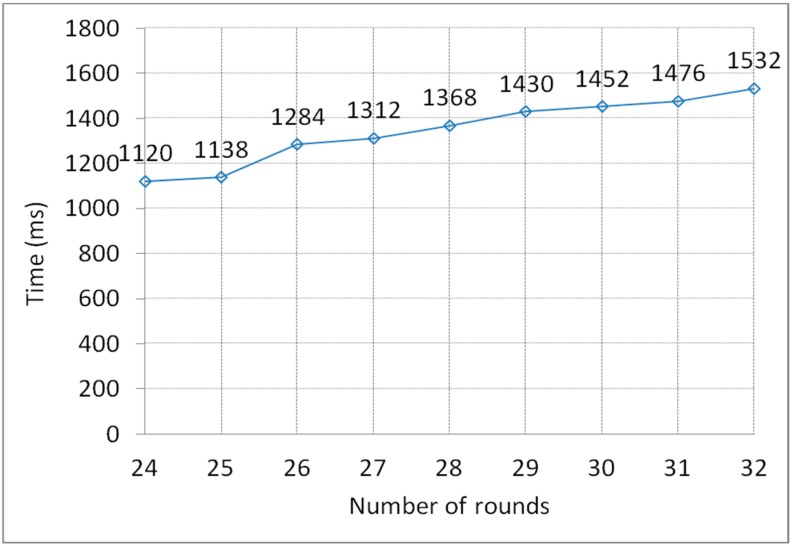
Experimental results of the performance test on Intel iMote.

The size of all T-Box lookup tables, matrixes and *α* values with respect to the number of rounds are illustrated in Figure 5. There are some frequently used operations in the encryption algorithm, such as T-Box table lookup, multiplication table lookup, (single) bit addition and byte addition. The overall numbers of these operations with respect to the number of rounds are illustrated in Figure 6. The performance of the encryption algorithm on Intel iMote with respect to the number of rounds is shown in Figure 7.

### 5.2. A Strong Version Using Non-Standard S-Boxes

In this sub-section, we discuss how to further improve the security of white-box SMS4. The basic idea is to use non-standard S-Boxes in the white-box implementation.

Instead of using Equation (8), we provide a new definition of substitution transformation $S_{i,j}$ as follows.

(20) $$S_{i,j}:GF{\left(2\right)}^{8}\to GF{\left(2\right)}^{8},x\mapsto \Lambda_{i}\left(\Theta_{i,j}\left({\Lambda_{i}}^{-1}\left(x\right)\right)\right)$$

where $\Theta_{i,j}$ is a randomly generated 8-bit to 8-bit permutation.

Consequently, let $x\in GF{\left(2\right)}^{8},y\in GF{\left(2\right)}^{32}$, the new T-Box lookup table with index <*i*,*j*>, *i.e*., $TBox_{i,j}$, is defined by Equation (23).

(21) $$y=TBox_{i,j}\left(x\right)=\left({\left(\Lambda_{i}||\Lambda_{i}||\Lambda_{i}||\Lambda_{i}\right)}^{-1}\left(\left(S_{i,j}\left(K_{i,j}+x\cdot E_{i,j}\right)+\alpha_{i,j}\right)\cdot M_{i,j}\right)\right)\cdot \Delta_{i+4}$$

The advantage of this improvement is that there are $2^{8}!$ possible constructions for $\Theta_{i,j}$. Hence, the implementation can achieve a higher security level. Because these S-Boxes are encapsulated in T-Boxes lookup tables, they do not involve extra costs in the process of encryption. However, in the process of decryption, the cost of using these random S-Boxes is that we have to store 128 S-Boxes instead of one in the original version. The decryption algorithm would need nearly $2^{8}\times 8\times 128=2^{18}$ bits (32 KB) extra static data. Note that introducing random S-Boxes may interfere with the black-box security properties of the resulting implementation of SMS4. Hence, we should use the standard number of rounds or even a few more rounds in this version of implementation. Furthermore, these S-Boxes are unknown to attackers. This would probably bring about significant difficulty to black-box cryptanalysis because widely used black-box analysis techniques, such as differential analysis and linear analysis, usually suppose that the only unknown factor of an encryption algorithm is the cryptographic key. 

## 6. Comparisons with Other Methods

**Figure 8:**
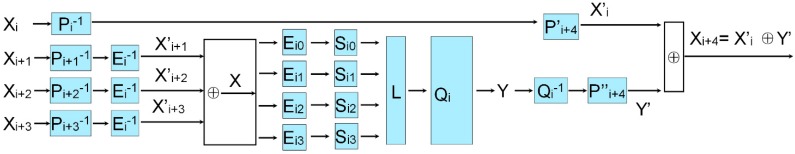
The structure of a round of Xiao *et al*.’s white-box SMS4.

We first compare our white-box SMS4 with another white-box SMS4 proposed by Xiao *et al*. in [10]. A round of the white-box SMS4 in [10] consists of three parts. We merge these parts together and connect them in sequence according to the encryption process to illustrate the structure of a round of Xiao *et al.*’s white-box SMS4 in Figure 8. Outside of T-Boxes, there are five affine 32-bit to 32-bit components in each round, *i.e*., ${{\text{P}}_{\text{i}}}^{-1}◦{\text{P′}}_{\text{i+4}}{{\text{, P}}_{\text{i+1}}}^{-1}◦{{\text{E}}_{\text{i}}}^{-1}{{\text{, P}}_{\text{i+2}}}^{-1}◦{{\text{E}}_{\text{i}}}^{-1}{{\text{, P}}_{\text{i+3}}}^{-1}◦{{\text{E}}_{\text{i}}}^{-1}{{\text{, Q}}_{\text{i}}}^{-1}◦{\text{P″}}_{\text{i+4}}$. Compared with our white-box SMS4, there are four 32-bit to 32-bit linear components in each round plus a 32-bit binary string outside of T-Boxes. Furthermore, in this paper, the diffusion transformations and the substitution transformations which are encapsulated in T-Boxes use a non-standard form after the “dual cipher” transformation. In the strong version, randomly generated S-Boxes are used to construct corresponding T-Boxes.

Moreover, in Table 2, we list the total size of the lookup tables of various white-box ciphers implementations in the second column, the efficiency in the third column, and the security in the fourth column. “Unknown” means that it is unknown whether there exists an effective attack.

**Table 2:** A comparison of white-box encryption algorithms.

Algorithm	Total Size of the Lookup Tables	Efficiency		Attack
		Table Lookup and XOR	Matrix Multiplication	
White-box DES [1]	4.5 MB	192	0	in [2,3,4]
White-box DES [5]	2.3 MB	384	0	in [3,4]
White-box AES [6]	752 KB	3104	0	in [7,8]
White-box AES [9]	20502 KB	120	11 (256 × 256)	in [10]
White-box AES [11]	752 KB	3104	0	in [12]
White-box SMS4 [13]	148.625 KB	96	160 (32 × 32)	in [15]
The proposed white-box SMS4 algorithm	144.125 KB	372	128 (32 × 32)	Unknown
The proposed aggressive white-box SMS4 algorithm	108.1 KB	264	96 (32 × 32)	Unknown
The proposed strong white-box SMS4 algorithm	144.125 KB	372	128 (32 × 32)	Unknown

As previously mentioned, while running a white-box encryption algorithm on a large block of data, the encryption speed can be reduced by using techniques introduced in [28]. Hence, distinctions in speed among various algorithms are not obvious when they are applied to a rather large data block.

## 7. Security against White-Box Attacks and Side-Channel Attacks

### 7.1. Threat Models and the Crux of Secure Implementations

**Figure 9:**
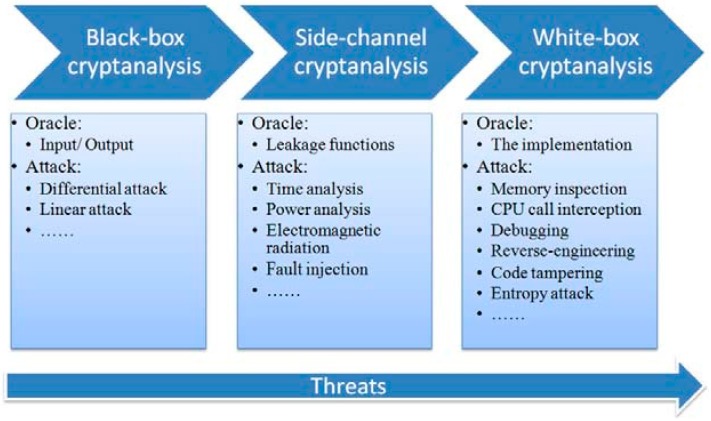
Attack models.

Before the discussion on security of the proposed white-box encryption algorithms, we briefly review three main attack/threat models capturing the capabilities of an adversary to attack cryptosystems [37]. The first one is the black-box model. It is a traditional attack model in which an adversary has only access to the functionality of a crypto system. The second one is the grey-box model, which refers to a model in which a leakage function is present. In such an attack context, the adversary can deploy side-channel cryptanalysis techniques. Due to the large variety of leakage functions, several grey-box models can be defined. The third one is the white-box model in which the adversary has total visibility of the software implementation of the cryptosystem and has full control over its execution platform. One could refer to the white-box model as the worst-case model. In contrast to grey-box models, it is impossible for an adversary not to comply with the model. The white-box model is used to analyze algorithms that are running in a non-trustable environment, in which applications are subject to attacks from the execution platform. Threats and cryptanalysis techniques in the three models are illustrated in Figure 9.

The main purpose of the proposed algorithms is to enable (implementations of) encryption algorithms securely running in WBACs, *i.e*., in the white-box model. As by-products, they are also secure against side-channel attacks. 

Next, we introduce the crux of how the proposed algorithms could resist against various white-box attacks and side-channel attacks in general. The main purpose of white-box attacks and side-channel attacks is to extract the cipher key from an implementation of an encryption algorithm. So, we focus on the components that contain information related to the round keys, *i.e*., lookup tables corresponding to T-Boxes.

Recall that these lookup tables only provide input/output of the following function:

(22) $$y=TBox_{i,j}\left(x\right)=\left({\left(\Lambda_{i}||\Lambda_{i}||\Lambda_{i}||\Lambda_{i}\right)}^{-1}\left(\left(S_{i}\left(K_{i,j}+x\cdot E_{i,j}\right)+\alpha_{i,j}\right)\cdot M_{i,j}\right)\right)\cdot \Delta_{i+4}$$

The secret mixing transformations $\Lambda_{i}$ and $\Delta_{i+4}$ are randomly selected from large sets, respectively. Furthermore, the linear transformation $L_{i,n}$ is implemented by multiplying a composition of a series of matrices given by (23).

(23) $$L_{i,n}=\left(\cdot {E_{i}}^{-1}\right)◦\left(\Lambda_{i}||\Lambda_{i}||\Lambda_{i}||\Lambda_{i}\right)◦\left(\cdot {\Delta_{i+n}}^{-1}\right)$$

Thus, it is hard for an attacker to deduce the concrete matrix corresponding to either Λ or Δ.

Furthermore, in the strong version, the functionality of each S-Box $S_{i}$ is randomly generated. This would bring significant difficulty to attackers since the linear equivalence (LE) algorithm and the affine equivalence (AE) algorithm are not applicable, where LE and AE are powerful cryptanalysis tools [21] which have been directly used or modified to break several white-box encryption algorithms successfully such as in [11,12,13].

Besides security matrices which are given in Section 4.1, security of a symmetric white-box encryption algorithm is verified by checking whether it is secure against related known attacks. This is similar to the case in the community of classical (black-box) symmetric cryptography. In the next subsection, the security of proposed algorithms against known attacks is investigated. 

In terms of side-channel cryptanalysis, they are not as powerful as the attacks in WBACs since leakage functions are restricted. Strictly, possible side-channel attacks consist of a subset of all possible attacks in the white-box model. Even though, how the proposed algorithms defeat side-channel attacks against (normal implementations of) SMS4 are briefly discussed at the end of section. 

Admittedly, a practical symmetric encryption algorithm, especially in the white-box model, usually could not find a strict security proof that reduces the breaking of an encryption algorithm into solving a computational infeasible mathematical problem. This would enable the authors to explore white-box encryption algorithms with a more complete theoretical foundation in future.

### 7.2. Against known White-Box Attacks 

Several attacks against white-box cryptography have been proposed. We briefly analyze these attacks in Table 3. In this table, “Direct Applicability” means that the attack technique can be used in attacking this algorithm without modification and “Potential Threat” means that the attack technique can probably be used to break this algorithm after slight modification. Moreover, <1>, <2> and <3> denote the algorithms proposed in Section 3, Section 5.1 (the aggressive white-box SMS4 algorithm) and Section 5.2 (the strong white-box SMS4 algorithm), respectively.

**Table 3:** Attacks against white-box cryptography.

Attack	Target	Base Algorithm	Direct Applicability			Potential Threat		
			<1>	<2>	<3>	<4>	<5>	<6>
[2]	[1]	DES	No	No	No	No	No	No
[3]	[5]	DES	No	No	No	No	No	No
[4]	[5]	DES	No	No	No	No	No	No
[7]	[6]	AES	No	No	No	No	No	No
[16]	white-box implementation for any SLT network cipher (using the design approach in [6])	SLT network cipher	No	No	No	No	No	No
[10]	[9]	AES	No	No	No	Yes	Yes	No
[15]	[13]	SMS4	No	No	No	Yes	Yes	No

As we have listed in the above table, two attack techniques, *i.e*., [13,23], are potentially threats to the first two proposed white-box encryption algorithms. So, we estimate the security of the first two proposed algorithms by analyzing how to break them based on techniques that are used in [13] or [23].

A toolbox presented in [21] is used by De Mulder *et al*. [13] to break [8] with a work factor of about 2^32^. The toolbox is presented by Biryukov *et al.* based on invariant properties of permutations (S-boxes) under the action of groups of linear or affine mappings. The toolbox provides efficient algorithms for solving the linear equivalence problem and the affine equivalence problem for arbitrary permutations (S-boxes). For a pair of n×n-bit permutations, the complexity of the affine equivalence algorithm is O(${\text{n}}^{3}2^{\text{2n}}$). The affine equivalence algorithm is efficient and allows studying affine equivalences for bijective S-boxes of all popular sizes (it is efficient up to *n* less than 32).

Based on [13] and [21], we design an attack that can be a potential threat against the first two white-box implementations as follows:

(1) Obtain leaked information about the linear input encoding.
(2) Find the desired linear equivalence and obtain the full linear input encoding.
(3) Extract a 32-bit round key.
(4) Extract four consecutive rounds and obtain the cryptographic key.
(5) Extract the external input and output encodings.

A conservative estimation of the work factor of getting a 32-bit round key is in Equation (24).

(24) $$4\times n^{3}\times 2^{2n}\times 2^{\log_{2}9360}\times 2^{8}>2^{2+9+16+13+8}=2^{48}$$

Hence, the work factor of extracting four consecutive rounds and obtaining the cryptographic key is greater than $2^{48}\times 4=2^{50}$. In practice, the work factor of breaking our first two algorithms by using this process may be much higher.

Moreover, Lin *et al.* proposed an efficient attack and explained in detail how to extract the round key embedded in the white box SMS4 implementation in [23]. We summarize the attack process as follows.

(1) Combine parts 2 and 3 of a round with part 1 of the next round and eliminating tabulating encodings between these two consecutive rounds.
(2) Recover the linear part of each affine transformation.
(3) Apply differential analysis to S-Boxes.
(4) Recover the constant part of each affine transformation by solving equations.
(5) Extract the round key from the implementation by solving matrix equations.

Lin and Lai claimed that their approach can extract the cryptographic key from a white-box SMS4 implementation with worst time complexity $2^{47}$.

The Λ transformation we use in this paper can provide a higher work factor. The overall work factor of applying Lin *et al*.’s attack against our white-box implementation is the product of the following three factors:

(1) $2^{47}$ to perform the basic attack process that is introduced above,
(2) $2^{13}(\approx 9230)$ to guess all the dual components in a round,
(3) $2^{5}$ for the total 32 rounds.

Thus, the security level of the proposed white-box SMS4 against a modified version of [23] may achieve

(25) $$2^{47+13+5}=2^{65}$$

Based on Equations (24) and (25), the security level of the proposed white-box SMS4 algorithm is assessed at about $2^{50}$.

The aim of our design is to make the size of implementation as small as possible in order to satisfy the restriction of computing in sensor nodes while protecting sensor nodes with time-limited security [20]. To achieve higher security and a longer protection time, we recommend that the strong version of white-box SMS4 in Section 5.2 should be used. Because there are there are $2^{8}!$ possible constructions for each S-Box, the work factor of a attack follows the idea of [23] would be about $2^{8}!$ times higher break than the normal white-box SMS4 in Section 3. Therefore, the strong version can be deployed in security sensitive scenarios because it is immune from attack techniques in [13] and [23] by using randomly generated secret S-Boxes.

### 7.3. Against known Side-Channel Attacks

Side-channel attack was first introduced by Kocher in 1996, using the information from the timing behavior. Since then, many other side-channels have been investigated, for example, power, electromagnetic emanation, fault injection and acoustic, *etc.* The context of side-channel attacks falls in the grey-box attack model, in which attackers are enhanced with the possibility to exploit physical leakages. Similar to white-box cryptanalysis, side-channel cryptanalysis utilizes exploitable vulnerability of a cryptosystem, not from a theoretical point of view, but from the implementation itself.

Suppose we execute a standard implementation of SMS4, the leakage of a small data fragment or a small set of information can already suffice to extract the cipher key. This remains true if we store the complete set of round keys instead of the main cipher key because an adversary can easily derive the cipher key from any round key. Otherwise, suppose that we implement SMS4 through a proposed white-box implementation instead of using a standard black-box implementation, such an implementation (*i.e*., a white-box encryption algorithm) is much larger and more complex than the black-box one. Furthermore, if an adversary has only part of the implementation, he or she will typically have difficulty deriving an implementation with the same functionality as the white-box implementation. To satisfy this condition, it must be difficult for an adversary to extract the key hidden in a white-box implementation from only part of this implementation. Therefore, in general, if we use a white-box implementation (instead of a standard implementation), an attacker typically has to derive much more data to obtain the implemented cryptographic functionality.

Concretely, why the proposed algorithms can defeat proposed side-channel attacks against (normal implementations of) SMS4 is briefly introduced as follows.

Li, Gu and Wang [38] studied the security of the contracting unbalanced Feistel networks structure against differential fault analysis (DFA) and showed that the 128-bit cipher key of a standard implementation of SMS4 can be recovered by 20 and four faulty ciphertexts. However, in a white-box implementation, the mathematical relationship between the inner states is secret. Moreover, the whole encryption process is also protected by secret external encodings. Hence, these attacks do not work on white-box implementations.

**Figure 10:**
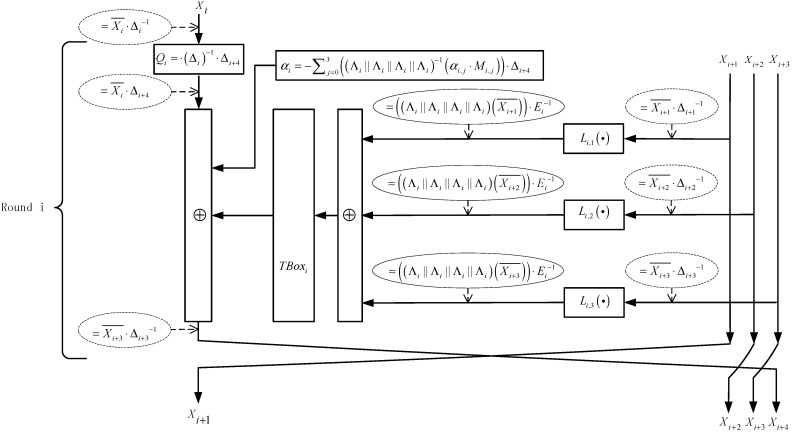
Non-standard states in the process of a white-box SMS4.

It was demonstrated in [39] that multi-process sharing cache space feature and SMS4 lookup table structure determine that SMS4 is vulnerable to cache timing attack, and about 80 samples are enough to recover the full 128-bit SMS4 key during both the first four rounds attack and last four round of an attack. A power analysis method for SMS4 to reduce the diffusion by chosen plaintext was proposed in [40]. The method can, in an orderly manner, acquire the first four rounds of key, and determine the master key of a 128-bit algorithm according to the key expansion algorithm. In the proposed algorithms, each key-dependent operation combined with the consequently S-Box lookup operation is embedded in a T-Box with randomly generated input/output masks. Therefore, in running the algorithms, the time and energy are mainly decided by the input (suppose *x*), rather than the T-Box lookup table because the output of a T-Box is obtained by fetching the *x*-th item in an array corresponding to the T-Box. Besides, as shown in Figure 10, when running a white-box SMS4 algorithm, the input and output of a component do not equal “standard states” in the corresponding normal implementation of SMS4 because they are multiplied by secret random matrices. Note that in Figure 10, mathematical descriptions of inner states are provided in ellipses where the symbol $\bar{X_{i}}$ denotes the value of a “standard state” in the normal implementation corresponds to the value of “non-standard state” $X_{i}$ in a white-box implementation.

## 8. Conclusions and Future Work

A class of lightweight white-box symmetric encryption algorithms against node captures for protecting sensor networks has been proposed in this paper. The first algorithm, which was proposed in IEEE WCNC 2014, is a slightly improved white-box SMS4. The second and the third ones are further improved based on the first one. Specifically, the second one is an aggressive white-box encryption algorithm that intends to acquire higher efficiency by reducing the number of rounds to at least 24. The third one is a strong white-box encryption algorithm that intends to acquire higher security against white-box cryptanalysis by using distinct randomly-generated S-Boxes rather than the fixed standard S-Box. The first two white-box encryption algorithms are capable of providing time-limited security for sensor nodes. The strong white-box SMS4 encryption algorithm is immune from all known attacks and their potential modifications against SMS4. Hence, it is expected to provide a much longer protection time. The proposed algorithms can serve as countermeasures against the threat of key exposure in the event of node capture. Moreover, they can also serve as countermeasures against a variety of side-channel attacks such as fault analysis, electromagnetic analysis and power analysis.

In terms of future work, we will explore novel approaches for designing white-box encryption algorithms with higher speed, smaller size, and a more complete theoretical foundation.
